# The bioluminescent *Listeria monocytogenes* strain Xen32 is defective in flagella expression and highly attenuated in orally infected BALB/cJ mice

**DOI:** 10.1186/1757-4749-5-19

**Published:** 2013-07-15

**Authors:** Silke Bergmann, Manfred Rohde, Klaus Schughart, Andreas Lengeling

**Affiliations:** 1 Department of Infection Genetics, Helmholtz Centre for Infection Research & University of Veterinary Medicine Hannover, Braunschweig D-38124, Germany; 2 Department of Medical Microbiology, Helmholtz Centre for Infection Research, Braunschweig D-38124, Germany; 3 University of Tennessee Health Science Center, Memphis, TN, USA; 4 Infection and Immunity Division, The Roslin Institute and R(D)SVS, University of Edinburgh, Easter Bush Veterinary Campus, Edinburgh EH25 9RG, UK

**Keywords:** Listeriosis, Flagella, Mouse infection model, Bioluminescent imaging

## Abstract

**Background:**

*In vivo* bioluminescence imaging (BLI) is a powerful method for the analysis of host-pathogen interactions in small animal models. The commercially available bioluminescent *Listeria monocytogenes* strain Xen32 is commonly used to analyse immune functions in knockout mice and pathomechanisms of listeriosis.

**Findings:**

To analyse and image listerial dissemination after oral infection we have generated a murinised Xen32 strain (Xen32-mur) which expresses a previously described mouse-adapted internalin A. This strain was used alongside the Xen32 wild type strain and the bioluminescent *L. monocytogenes* strains EGDe-lux and murinised EGDe-mur-lux to characterise bacterial dissemination in orally inoculated BALB/cJ mice. After four days of infection, Xen32 and Xen32-mur infected mice displayed consistently higher rates of bioluminescence compared to EGDe-lux and EGDe-mur-lux infected animals. However, surprisingly both Xen32 strains showed attenuated virulence in orally infected BALB/c mice that correlated with lower bacterial burden in internal organs at day 5 post infection, smaller losses in body weights and increased survival compared to EGDe-lux or EGDe-mur-lux inoculated animals. The Xen32 strain was made bioluminescent by integration of a *lux*-*kan* transposon cassette into the listerial *flaA* locus. We show here that this integration results in Xen32 in a *flaA* frameshift mutation which makes this strain flagella deficient.

**Conclusions:**

The bioluminescent *L. monocytogenes* strain Xen32 is deficient in flagella expression and highly attenuated in orally infected BALB/c mice. As this listerial strain has been used in many BLI studies of murine listeriosis, it is important that the scientific community is aware of its reduced virulence *in vivo.*

## Findings

### Background

Bioluminescent *in vivo* imaging (BLI) of *Listeria monocytogenes* infections in mice has generated several new insights into the pathogenesis of listeriosis. For example, the now commercially available *Listeria monocytogenes* strain Xen32 was first used to demonstrate that the gallbladder is an important organ reservoir of listerial replication and pathogen shedding [1-3]. Since then the Xen32 listerial strain has been used in multiple studies as a tool to study *Listeria* directed immune mechanisms in knockout mice [4] and kinetics of *L. monocytogenes* dissemination to target organs of listeriosis such as the bone marrow [5]. More recently, the bioluminescent Xen32 strain has also been used to study transplacental transmission of *L. monocytogenes* in fetal listeriosis [6,7].

The aim of this study was to use the bioluminescent *L. monocytogenes* strain Xen32 in an oral mouse listeriosis model to analyse the dissemination of the pathogen from the intestine to internal organs. To enable efficient transmission of this listerial strain through the murine gut mucosa we used our previous approach of murinisation to optimise the binding of the listerial surface protein internalin A (InlA) to the murine E-cadherin host receptor [8].

### Material and methods

For oral inoculation of female, 9-10 weeks old BALB/cJ mice (Harlan Winkelmann, Borchen, Germany) we used our previously published mouse infection model [9]. All experiments were conducted according to German animal welfare regulations after approval from the Niedersächsisches Landesamt für Verbraucherschutz und Lebensmittelsicherheit (LAVES) as the local authority. The *Listeria monocytogenes* strains EGDe-lux (Lmo-EGDe-lux) and *L. monocytogenes* EGDe-InlA-mur-lux (Lmo-EGDe-mur-lux) have been described previously [10]*. L. monocytogenes* Xen32 was purchased from Perkin Elmer (Rodgau, Germany) and genetically modified for expression of a mouse-adapted InlA as previously described [8]. Analysis of bacterial organ counts (colony forming units, CFU) was performed as described in Bergmann et al. [9]. BLI images were obtained using an IVIS 200 imaging system (CaliperLS) with integration time of 3 min (Xen32 strains) or 4 min (EGDe strains) at a binning of 8 and F/stop of 1. Photon flux was quantified by using the Living Image 3.1 software (CaliperLS). To assess general growth characteristics of the different *L. monocytogenes* strains, growth curves were performed as previously described [11]. Luminescence was measured by quantifying photon flux of 0.5 ml culture samples from a 50 ml logarithmic *L. monocytogenes* culture at indicated timepoints on the IVIS 200 imaging system (5 sec integration time, binning of 8 and F/stop of 1). Genomic *flaA* fragments from *L. monocytogenes* Xen32 were amplified with *flaA* forward primer (5′-AGAGAAGTCTTTTCTAAACCGAATGTAGGA-3′) and *flaA* reverse primer (5′-CTAAGGGTAAACAATGTTCGATAAATG-3′), sequenced and analysed with MacVector 11.0.2 (MacVector Inc., Cambridge, UK). For analysis of flagella expression, listerial strains were grown overnight in BHI medium at 24°C and negatively stained with 2% uranyl acetate and examined in a Zeiss TEM910 at 80 kV. Cell invasion assays with the human colorectal epithelial cell line Caco-is deficient in flagella expression 2 (ATCC HTB-37) and the murine colon carcinoma cell line CT26 (ATCC CRL-2639) were performed as previously described [8]. Statistical analysis of CFU data was performed using the Mann–Whitney U non-parametic test and the GraphPad Prism 5 (version 5.01) analysis software (GraphPad Software Inc.). Survival curves were statistically evaluated by Kaplan-Meier and Log- rank (Mantel-Cox) analyses.

### Results and discussion

**Figure 1:**
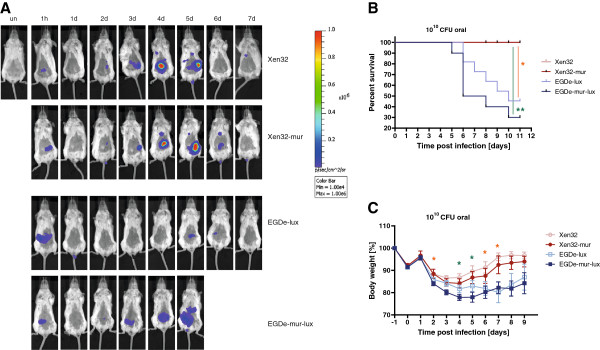
***Listeria monocytogenes*Xen32 strains show attenuated virulence*in vivo.*** BALB/cJ mice (n = 10) were intragastrically inoculated with 1 × 10^10^ CFU Lmo-Xen32, Lmo-Xen32-mur, Lmo-EGDe-lux or Lmo-EGDe-mur-lux. **(A)** Serial BLI of 1 representative mouse from one hour until 7 days post infection as described in Methods (see also Additional file 1: Figure S1 for reference). Colour bar indicates emitted light with 3 or 4 min integration time in photons/s/cm2/sr. un = uninfected control. **(B)** Survival rates of BALB/cJ mice (n = 10) after infection with 1 × 10^10^ CFU Lmo-Xen32, Lmo-Xen32-mur, Lmo-EGDe-lux or Lmo-EGDe-mur-lux. **(C)** Body weight loss of BALB/cJ mice infected with the same listerial strains and dosage as shown in **(A)** and **(B)**. Graphs demonstrated mean in percent (n = 10) with standard error. Statistical significance between murinised (green stars) and non-murinised (yellow stars) *Listeria* strains are indicated (*p < 0.05, **p < 0.01, non-parametric Mann–Whitney-*U*-test).

**Figure 2:**
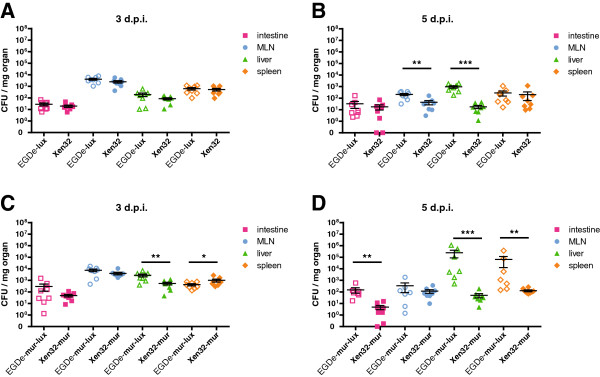
**Lmo-Xen32 strains show reduced organ burden after oral infection of BALB/c mice compared to Lmo-EGDe strains.** BALB/cJ mice (n = 8) were infected with 5 × 10^9^ CFU Lmo-Xen32, Lmo-Xen32-mur, Lmo-EGDe-lux or Lmo-EGDe-mur-lux. At indicated time points bacterial organ loads were determined in the small intestine, mesenteric lymph nodes, liver and spleen (n = 8). 3 d.p.i **(A, C)** and 5 d.p.i. **(B, D)** indicate 3 or 5 days post infection, respectively. Graphs demonstrated mean with standard error of bacterial load in inner organs. Statistical significance between Xen32 and EGDe strains are indicated (*p < 0.05, **p < 0.01, ***p < 0.001, non-parametric Mann–Whitney-*U*-test).

**Figure 3:**
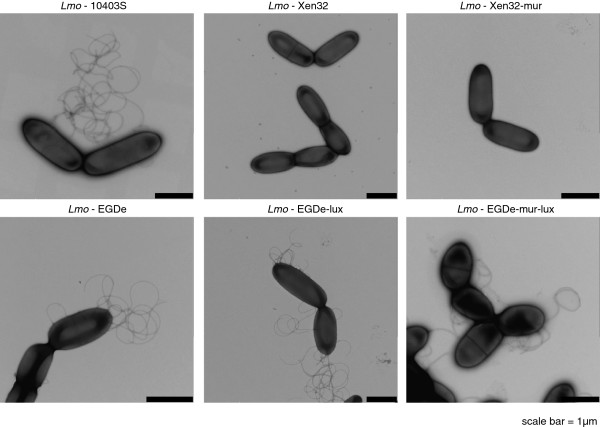
***L. monocytogenes*Xen32 strains are flagella deficient.** Transmission electron microscopy of Lmo-Xen32, Lmo-Xen32-mur, Lmo-10403S, Lmo-EGDe-lux, Lmo-EGDe-mur-lux and Lmo-EGDe. Scale bars indicate 1 μm.

The *L. monocytogenes* strain Xen32 was originally generated by screening a *lux*-*kan* transposon integration library of the parental strain *L. monocytogenes* strain 10403S for high levels of bioluminescence [1]. Xen32, also named clone 2C, was selected after *in vivo* evaluation of strong photon emission in infected BALB/c mice [1]. To further optimise this strain for oral infection challenge in mice we generated an isogenic mutant by replacing the wild type *inlA* with *inlA*^*S192N-Y369S*^ as previously described [8]. The new murinised *L. monocytogenes* strain was called Xen32-mur and the introduced mutations in the *inlA* locus validated by sequencing and Western blot analysis of InlA and internalin B protein expression (data not shown). All *L. monocytogenes* strains were profiled for *in vitro* growth in BHI media and emitted levels of luminescence. While no differences in *in vitro* growth rates were found between the listerial strains, both Xen32 strains showed higher levels of bioluminescence compared to the EGDe strains (Additional file 1: Figure S1). BALB/cJ mice were intragastrically infected with 10^10^ CFU of *L. monocytogenes* (Lmo) Xen32, Lmo-Xen32-mur, Lmo-EGDe-lux, or Lmo-EGDe-mur-lux and analysed by BLI for a time period of 9 days post infection (d.p.i.). After 4 days of infection, Lmo-Xen32 and Lmo-Xen32-mur infected mice displayed high levels of light emission in abdominal regions (Figure 1A and Additional file 2: Figure S2). The measured level of bioluminescence in these regions was about 10-fold higher in Lmo-Xen32 infected mice compared to Lmo-EGDe-lux infected mice and 5-fold more intense in Lmo-Xen32-mur infected animals compared to Lmo-EGDe-mur-lux infected animals. The intense BLI signals in Lmo-Xen32 and Lmo-Xen32-mur infected mice remained high for one additional day (5 d.p.i.) and decreased then until no further signals remain detectable at 9 d.p.i. (Additional File 2: Figure S2). Surprisingly despite these high levels of bioluminescence, Lmo-Xen32 and Lmo-Xen32-mur infected mice lost less body weight (Figure 1B) and had significantly lower mortality rates compared to Lmo-EGDe-lux and EGDe-mur-lux infected mice (Figure 1C). In contrast, infection with both EGDe listerial strains resulted in more drastic losses in body weights and reduced survival after 5 d.p.i. (Figure 1C). To further analyse differences in strain virulence we determined bacterial organ loads after 3 and 5 d.p.i. with 5 × 10^9^ CFU bacteria. At 5 d.p.i., Lmo-EGDe-lux infected mice had about 10-fold higher CFU loads in mesenteric lymph nodes and 100-fold higher bacterial organ loads in the liver compared to Lmo-Xen32 infected mice (Figure 2B). Higher CFU loads in Lmo-EGDe-mur-lux infected mice were also detected at 3 d.p.i. in the liver compared to Lmo-Xen32-mur infected mice (Figure 2C). These became more prominent at 5 d.p.i. with about 10-fold higher CFU counts in the intestine and about 100-fold and 1000-fold higher organ loads in spleen and liver, respectively, in Lmo-EGDe-mur-lux infected mice compared to Lmo-Xen32-mur infected animals (Figure 2D). Thus, the higher mortality observed in Lmo-EGDe-lux and Lmo-EGDe-mur-lux infected mice correlated with increased organ loads compared to Lmo-Xen32 and Lmo-Xen32-mur infected mice and not with the level of detected bioluminescence. The Lmo-Xen32 strains appeared to emit *in vivo* higher light levels compared to the EGDe listerial strains. However, this was not related to virulence in our mouse infection model. The LD_50_ for Lmo-Xen32 in BALB/c mice has been described to be four times higher compared to the parental *L. monocytogenes* strain 10403S [1]. Because the parental *L. monocytogenes* strains 10403S and EGDe are known to have similar LD_50_ dosages in BALB/c mice [12-14] we were surprised by the big difference in listeriosis outcome that we observed in this study between mice that were infected with derivates of the 10403S and EGDe strains. In Xen32 the lux operon was integrated via transposon insertion into the flagellin encoding *flaA* locus [1]. The consequences of this integration have so far not been analysed. Therefore, we sequenced *flaA* in Lmo-Xen32 and found that the insertion of the *lux*-*kan* transposon cassette caused a frameshift mutation which generates an amber stop codon (TAG) 80 bp downstream of the methionin start codon (Additional file 3: Figure S3). This mutation would result in the expression of a truncated, non-functional flagellin fusion protein of 27 amino acids instead of the full-length flagellin protein of 287 amino acids. Indeed, when we examined the parental listerial strains Lmo-10403S, Lmo-EGDe, and the bioluminescent strains Lmo-EGDe-lux and EGDe-mur-lux with transmission electron microscopy we found that all these strains clearly expressed peritrichous flagella. In contrast, both Xen32 strains were found to be completely flagella-deficient (Figure 3A). To assess whether the Xen32 mutation influence invasion into human Caco-2 and murine CT26 cells, we performed Gentamicin-protection-invasion assays with Lmo-Xen32, Lmo-Xen32-mur, Lmo-EGDe-lux and Lmo-EGDe-mur-lux. Lmo-EGDe-mur-lux invaded murine CT26 cells and to a lesser extent also human Caco-2 cells more efficiently than Lmo-EGDe-lux (Additional file 4: Figure S4A, S4C) in line with previous results [8,10]. No such differences were detectable between the Lmo-Xen32 and Lmo-Xen32-mur strains (Additional file 4: Figure S4B, S4D) suggesting that flagella are required for optimal invasion of CT26 and Caco-2 cells as previously demonstrated by O’Neil and Marquis [15].

The possible roles of flagella in listeriosis pathogenesis are diverse. They include host cell adhesion and invasion, injection of virulence factors and initiation and modulation of host inflammation through recognition of flagellin by TLR5 and inflammasome receptors [16-19]. Previous studies have reported different effects of flagella deficiency on the pathogenesis of oral *L. monocytogenes* infections *in vivo*. The virulence of flagellin deletion mutants or mutants with deficiency in flagella regulatory proteins was found to be either increased or decreased depending on the listerial strain that was used for genetic modification or the mouse infection model that was employed [15,17,18,20,21]. However, these studies have used *L. monocytogenes* strains for oral infection challenge that were not optimized for InlA-mediated recognition of the murine E-cadherin receptor. We report here that Lmo-EGDe-lux shows increased *in vivo* virulence after oral infection challenge when compared to flagella deficient Lmo-Xen32 and that this difference in virulence between EGDe and Xen32 strains becomes even bigger when both strains are murinised for InlA.

### Conclusions

The bioluminescent *L. monocytogenes* strain Xen32 is deficient in flagella expression and highly attenuated in virulence in an oral mouse infection model. Despite this attenuation in virulence, the *L. monocytogenes* strain Xen32 might be still a useful tool for *in vivo* imaging in experiments where sublethal or very mild infections are required (e.g. for example for phenotyping of highly susceptible or immunocompromised mouse strains). However, the scientific community should be aware that infections with *L. monocytogenes* Xen32 of wild type, immunocompetent mouse strains might result in smaller effects on host responses due to its *in vivo* virulence attenuation.

## Competing interests

The authors declare that they have no competing interests.

## Authors’ contributions

SB conducted all mouse infection experiments. MR did the transmission electron microscopy analysis of the different *L. monocytogenes* strains. KS contributed to the study design and coordination of experiments. AL designed experiments, analysed data and drafted the manuscript. All authors read and approved the final manuscript.

## Supplementary Material

Additional file 1: Figure S1 *In vitro* growth and luminescence profiles of Lmo-Xen32, Lmo-Xen32-mur, Lmo-EGDe-lux and Lmo-EGDe-mur-lux. Lmo-Xen32, Lmo-Xen32-mur, Lmo-EGDe-lux and Lmo-EGDe-mur-lux were grown in triplicates in BHI media and their growth curves and emitted levels of luminescence measured as described in Material and Methods. No major differences were detected in strain growth rates but Xen32 strains emitted higher levels of luminescence at indicated timepoints. Click here for file

Additional file 2: Figure S2 Bioluminescence Imaging of orally infected mice with 1 × 10^10^ CFU Lmo-Xen32, Lmo-Xen32-mur, Lmo-EGDe-lux and Lmo-EGDe-mur-lux. BALB/cJ mice were intragastrically infected with 1 × 10^10^ CFU Lmo-Xen32, Lmo-Xen32-mur, Lmo-EGDe-lux or Lmo-EGDe-mur-lux and the progress of infection was assessed by BLI for 9 days as described in Methods. Serial BLI data are shown for a set of 5 representative mice out of 10 for a time period of 9 days p.i.. Empty spaces indicate mice that succumbed to the infection or were euthanized for ethical reasons. The colour bar indicates photon emission with 3 or 4 min integration time in photons/s/cm^2^/sr. Click here for file

Additional file 3: Figure S3 Sequence of the *lux-kan* transposon integration site in the *flaA* locus of *Listeria monocytogenes* strain Xen32. Shown are the nucleotide and translated protein sequences. The insertion of the *lux*-*kan* transposon cassette results in a frameshift mutation with the generation of an amber stop codon (TAG) after 80 bp. Translated protein sequences of *flaA* are shown in blue, the frameshift protein translation is shown in red. The amber stop codon is underlined and depicted in bold red. Click here for file

Additional file 4: Figure S4 Invasion and intracellular growth of Lmo-Xen32, Lmo-Xen32-mur, Lmo-EGDe-lux and Lmo-EGDe-mur-lux. Confluent layers of Caco2 and CT26 cells were infected for 60 min with Lmo-Xen32, Lmo-Xen32-mur, Lmo-EGDe-lux and Lmo-EGDe-mur-lux. Extracellular bacteria were killed by gentamycin treatment (100 μg/ml). At indicated timepoints cells were washed with PBS and lysed with sterile water containing 0,2% Triton X-100. Intracellular bacteria were quantified by plating serial dilutions of cell lysates on BHI agar plates. Graphs demonstrated mean CFU values of triplicate growth assays for each strain with standard error. Statistical significance between strains is indicated (*p < 0.05, ***p < 0.001, non-parametric Mann–Whitney-*U*-test). Click here for file

## References

[B1] Hardy J Francis KP DeBoer M Chu P Gibbs K Contag CH Extracellular replication of *Listeria monocytogenes* in the murine gall bladder Science 2004 303 851 853 10.1126/science.1092712 14764883

[B2] Hardy J Margolis JJ Contag CH Induced biliary excretion of *Listeria monocytogenes* Infect Immun 2006 74 1819 1827 10.1128/IAI.74.3.1819-1827.2006 16495556 PMC1418634

[B3] Contag PR Bioluminescence imaging to evaluate infections and host response *in vivo* Meth Mol Biol 2008 415 101 118 10.1007/978-1-59745-570-1_6 18370150

[B4] Brandl K Plitas G Schnabl B DeMatteo RP Pamer EG MyD88-mediated signals induce the bactericidal lectin RegIII gamma and protect mice against intestinal *Listeria monocytogenes* infection J Exp Med 2007 204 1891 1900 10.1084/jem.20070563 17635956 PMC2118673

[B5] Hardy J Chu P Contag CH Foci of *Listeria monocytogenes* persist in the bone marrow Dis Model Mech 2009 2 39 46 10.1242/dmm.000836 19132117 PMC2615163

[B6] Hardy J Kirkendoll B Zhao H Pisani L Luong R Switzer A McConnell MV Contag CH Infection of pregnant mice with *Listeria monocytogenes* induces fetal bradycardia Pediatr Res 2012 71 539 545 10.1038/pr.2012.2 22314663

[B7] Poulsen KP Faith NG Steinberg H Czuprynski CJ Pregnancy reduces the genetic resistance of C57BL/6 mice to *Listeria monocytogenes* infection by intragastric inoculation Microb Pathog 2011 50 360 366 10.1016/j.micpath.2011.02.003 21320586 PMC3085720

[B8] Wollert T Pasche B Rochon M Deppenmeier S van den Heuvel J Gruber AD Heinz DW Lengeling A Schubert WD Extending the host range of *Listeria monocytogenes* by rational protein design Cell 2007 129 891 902 10.1016/j.cell.2007.03.049 17540170

[B9] Bergmann S Beard PM Pasche B Lienenklaus S Weiss S Gahan CG Schughart K Lengeling A Influence of Internalin A murinisation on host resistance to orally acquired listeriosis in mice BMC Microbiol 2013 13 90 10.1186/1471-2180-13-90 23617550 PMC3640945

[B10] Monk IR Casey PG Hill C Gahan CG Directed evolution and targeted mutagenesis to murinize *Listeria monocytogenes* internalin A for enhanced infectivity in the murine oral infection model BMC Microbiol 2010 10 318 10.1186/1471-2180-10-318 21144051 PMC3016325

[B11] Roberts AJ Williams SK Wiedmann M Nightingale KK Some *Listeria monocytogenes* outbreak strains demonstrate significantly reduced invasion, inlA transcript levels, and swarming motility *in vitro* Appl Environ Microbiol 2009 75 5647 5658 10.1128/AEM.00367-09 19581477 PMC2737929

[B12] Mackaness GB The Immunological Basis of Acquired Cellular Resistance J Exp Med 1964 120 105 120 10.1084/jem.120.1.105 25240018

[B13] Portnoy DA Jacks PS Hinrichs DJ Role of hemolysin for the intracellular growth of *Listeria monocytogenes* J Exp Med 1988 167 1459 1471 10.1084/jem.167.4.1459 2833557 PMC2188911

[B14] Busch DH Vijh S Pamer EG Coligan JE Animal model for infection with *Listeria monocytogenes* Current protocols in immunology 2001 Hoboken, USA: John Wiley & Sons, Inc Chapter 19: Unit 19.9 10.1002/0471142735.im1909s36 18432760

[B15] O’Neil HS Marquis H *Listeria monocytogenes* flagella are used for motility, not as adhesins, to increase host cell invasion Infect Immun 2006 74 6675 6681 10.1128/IAI.00886-06 16982842 PMC1698079

[B16] Hayashi F Smith KD Ozinsky A Hawn TR Yi EC Goodlett DR Eng JK Akira S Underhill DM Aderem A The innate immune response to bacterial flagellin is mediated by Toll-like receptor 5 Nature 2001 410 1099 1103 10.1038/35074106 11323673

[B17] Dons L Eriksson E Jin Y Rottenberg ME Kristensson K Larsen CN Bresciani J Olsen JE Role of flagellin and the two-component CheA/CheY system of *Listeria monocytogenes* in host cell invasion and virulence Infect Immun 2004 72 3237 3244 10.1128/IAI.72.6.3237-3244.2004 15155625 PMC415653

[B18] Bigot A Pagniez H Botton E Frehel C Dubail I Jacquet C Charbit A Raynaud C Role of FliF and FliI of *Listeria monocytogenes* in flagellar assembly and pathogenicity Infect Immun 2005 73 5530 5539 10.1128/IAI.73.9.5530-5539.2005 16113269 PMC1231047

[B19] Zhao Y Yang J Shi J Gong YN Lu Q Xu H Liu L Shao F The NLRC4 inflammasome receptors for bacterial flagellin and type III secretion apparatus Nature 2011 477 596 600 10.1038/nature10510 21918512

[B20] Grundling A Burrack LS Bouwer HG Higgins DE *Listeria monocytogenes* regulates flagellar motility gene expression through MogR, a transcriptional repressor required for virulence Proc Natl Acad Sci USA 2004 101 12318 12323 10.1073/pnas.0404924101 15302931 PMC514476

[B21] Way SS Thompson LJ Lopes JE Hajjar AM Kollmann TR Freitag NE Wilson CB Characterization of flagellin expression and its role in *Listeria monocytogenes* infection and immunity Cell Microbiol 2004 6 235 242 10.1046/j.1462-5822.2004.00360.x 14764107

