# Supplementary figures and images for: The bioluminescent Listeria monocytogenes strain Xen32 is defective in flagella expression and highly attenuated in orally infected BALB/cJ mice
Source: Gut Pathog. 2013 Jul 15;5:19. doi: 10.1186/1757-4749-5-19 (PMC3720536; doi:10.1186/1757-4749-5-19)

A

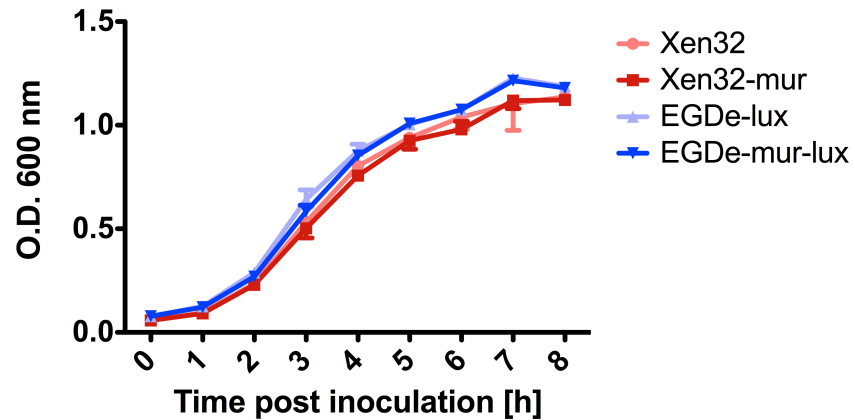

B

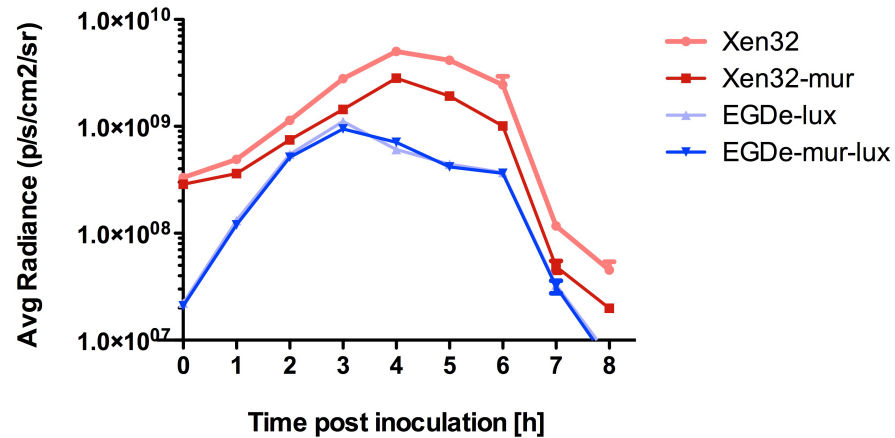

Supplement: Additional file 1: Figure S1 — In vitro growth and luminescence profiles of Lmo-Xen32, Lmo-Xen32-mur, Lmo-EGDe-lux and Lmo-EGDe-mur-lux. Lmo-Xen32, Lmo-Xen32-mur, Lmo-EGDe-lux and Lmo-EGDe-mur-lux were grown in triplicates in BHI media and their growth curves and emitted levels of luminescence measured as described in Material and Methods. No major differences were detected in strain growth rates but Xen32 strains emitted higher levels of luminescence at indicated timepoints. [file 1757-4749-5-19-S1.pdf]

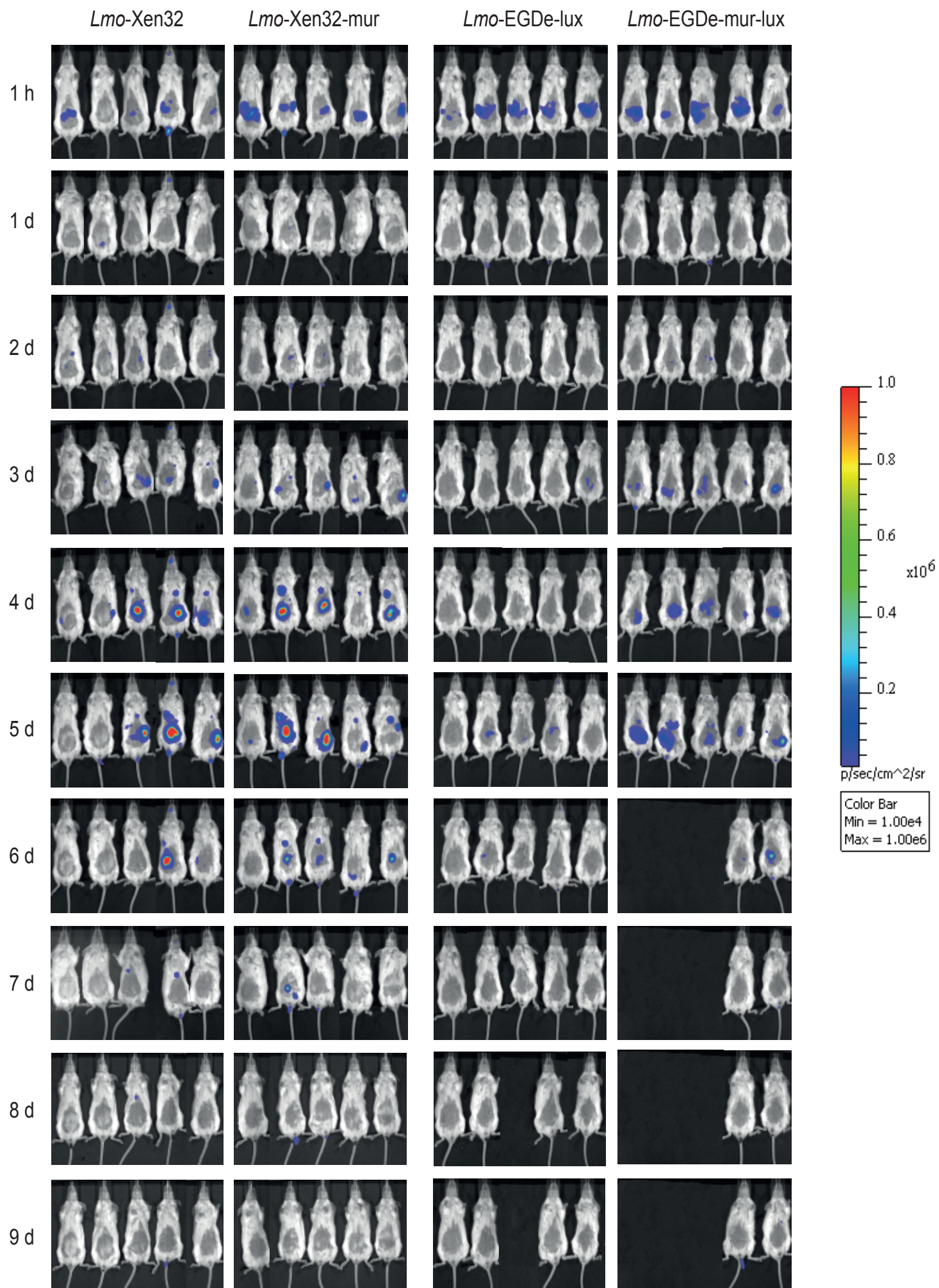

Supplement: Additional file 2: Figure S2 — Bioluminescence Imaging of orally infected mice with 1 × 10^10^ CFU Lmo-Xen32, Lmo-Xen32-mur, Lmo-EGDe-lux and Lmo-EGDe-mur-lux. BALB/cJ mice were intragastrically infected with 1 × 10^10^ CFU Lmo-Xen32, Lmo-Xen32-mur, Lmo-EGDe-lux or Lmo-EGDe-mur-lux and the progress of infection was assessed by BLI for 9 days as described in Methods. Serial BLI data are shown for a set of 5 representative mice out of 10 for a time period of 9 days p.i.. Empty spaces indicate mice that succumbed to the infection or were euthanized for ethical reasons. The colour bar indicates photon emission with 3 or 4 min integration time in photons/s/cm2/sr. [file 1757-4749-5-19-S2.pdf]

CT26

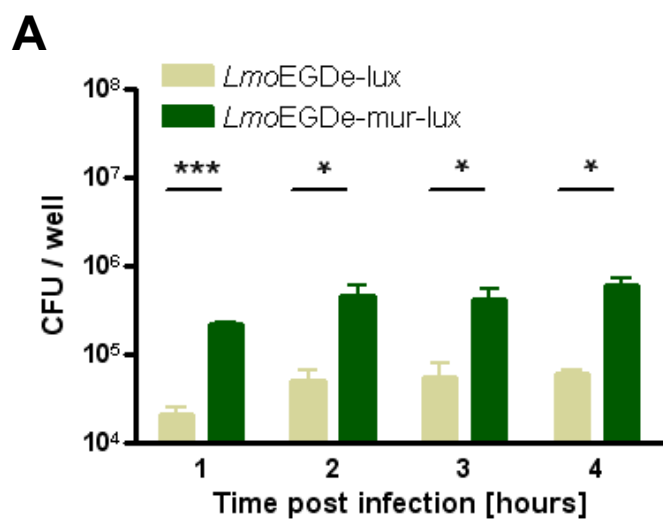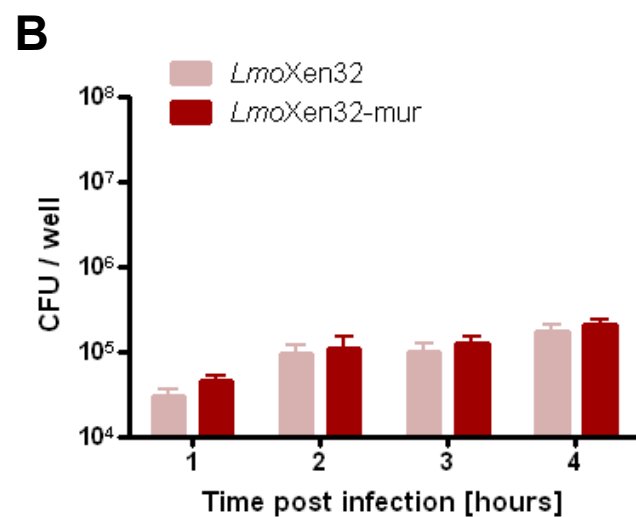

Caco-2

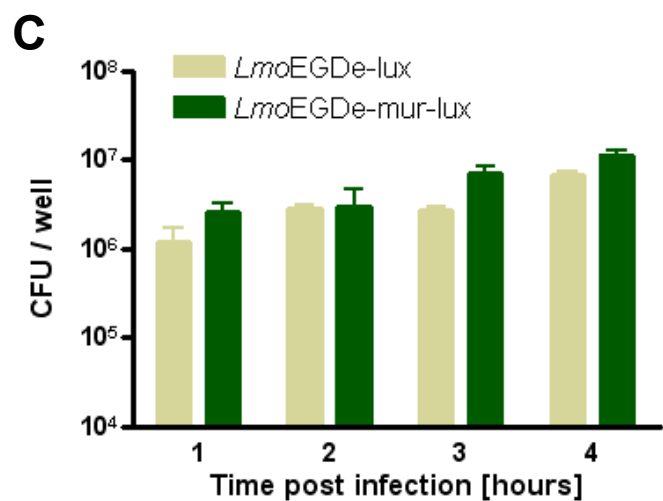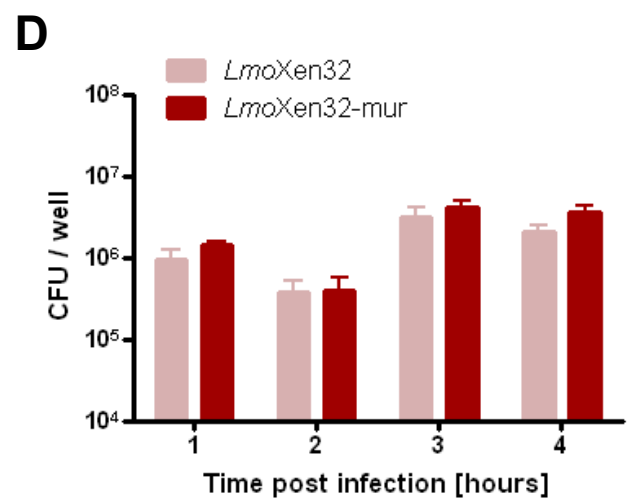

Supplement: Additional file 4: Figure S4 — Invasion and intracellular growth of Lmo-Xen32, Lmo-Xen32-mur, Lmo-EGDe-lux and Lmo-EGDe-mur-lux. Confluent layers of Caco2 and CT26 cells were infected for 60 min with Lmo-Xen32, Lmo-Xen32-mur, Lmo-EGDe-lux and Lmo-EGDe-mur-lux. Extracellular bacteria were killed by gentamycin treatment (100 μg/ml). At indicated timepoints cells were washed with PBS and lysed with sterile water containing 0,2% Triton X-100. Intracellular bacteria were quantified by plating serial dilutions of cell lysates on BHI agar plates. Graphs demonstrated mean CFU values of triplicate growth assays for each strain with standard error. Statistical significance between strains is indicated (*p < 0.05, ***p < 0.001, non-parametric Mann–Whitney-U-test). [file 1757-4749-5-19-S4.pdf]
