# Supplementary material for: The bioluminescent Listeria monocytogenes strain Xen32 is defective in flagella expression and highly attenuated in orally infected BALB/cJ mice
Source: Gut Pathog. 2013 Jul 15;5:19. doi: 10.1186/1757-4749-5-19 (PMC3720536; doi:10.1186/1757-4749-5-19)
Supplement: Additional file 3: Figure S3 — Sequence of the lux-kan transposon integration site in the flaA locus of Listeria monocytogenes strain Xen32. Shown are the nucleotide and translated protein sequences. The insertion of the lux-kan transposon cassette results in a frameshift mutation with the generation of an amber stop codon (TAG) after 80 bp. Translated protein sequences of flaA are shown in blue, the frameshift protein translation is shown in red. The amber stop codon is underlined and depicted in bold red. [file 1757-4749-5-19-S3.pdf]

Supplementary Figure S3

```
         10         20         30         40         50
ATGAAAGTAA ATACTAATAT CATTAGCTTG AAAACACAAG AATATCTTCG
TACTTTCATT TATGATTATA GTAATCGAAC TTTTGTGTTC TTATAGAAGC
 M  K  V   N  T  N  I   I  S  L   K  T  Q   E  Y  L  R
 >_____________________ORF flaA _____________________<

         60         70         80         90        100
AGTCAAGTCC AGACTCCTGT GTAAAATGAT CTAGTGGATC CTGCAGATGA
TCAGTTCAGG TCTGAGGACA CATTTTACTA GATCACCTAG GACGTCTACT
  V  K  S   R  L  L   C  K  M  I   *>
>____ORF frameshift mutation___<
```

Sequence of the lux-kan transposon integration site in the *flaA* locus of *Listeria monocytogenes* strain Xen32.

Shown are the nucleotide and translated protein sequences. The insertion of the lux-kan transposon cassette results in a frameshift mutation with the generation of an amber stop codon (TAG) after 80 bp. Translated protein sequences of *flaA* are shown in blue, the frameshift protein translation is shown in red. The amber stop codon is underlined and depicted in bold red.
